# The Therapeutic Value of the Rectum in the Treatment of Disease in Children

**Published:** 1909-01-23

**Authors:** Edmund Cautley

**Affiliations:** Physician to the Belgrave Hospital for Children and to the Metropolitan Hospital


### Hospital Clinics.

### THE THERAPEUTIC VALUE OF THE RECTUM IN THE TREATMENT OF DISEASE IN CHILDREN.

By EDMUND CAUTLEY, M.D., F.R.C.P., Physician to the Belgrave Hospital for Children and to the Metropolitan Hospital.

I have chosen this subject for a clinical lecture, for it is somewhat neglected in text-books, and insufficiently valued by many members of our profession. Probably it is partly due to æsthetic grounds, partly to lack of practical experience, that the rectum is not made use of in proportion to its importance as a channel for the administration of drugs and the treatment of disease in early life. It may be used for the following purposes:—

1. The introduction of water and food.
2. The reduction of the body temperature.
3. The treatment of collapse.
4. The treatment of local and general diseases, such as (*a*) pain and tenesmus, colic; (*b*) abdominal distension; (*c*) constipation; (*d*) colitis and other affections causing diarrhœa; (*e*) prolapse of the rectum; (*f*) thread-worms.
5. The introduction of drugs which for various reasons cannot be given by the mouth or subcutaneously.

First of all it is necessary to consider the method of irrigation of the colon, for it is upon this that the treatment of many diseases depends, and in others it is an important adjunct. I must also point out that rectal treatment includes absorption by the colon, for substances introduced into the rectum pass upwards into the colon as far as the cæcum and even further, in consequence of the reflex currents which are set up. Insoluble substances may even reach the stomach, so it is erroneous to suppose, as is often done, that the absorption of rectal feeds takes place invariably below the ileo-cæcal valve. Indeed, it is probable that cases, in which rectal feeding has proved unusually satisfactory, have been those in which the reflex currents have carried the food upwards into the small intestine. Another point to bear in mind is that the capacity for absorption varies in different subjects and with the nature of the nutriment offered, rather than with the percentage composition of the nutrient fluid.

Irrigation of the colon is necessary for cleansing purposes, as a preliminary to giving rectal feeds, to empty the bowels and wash away the residue of decomposing food left from previous meals. It should be done once in twenty-four hours, and is not required before each feed. It is valuable for washing away mucus, degenerated epithelium, bacteria, and toxic substances in inflammatory affections, for the relief of thirst, for the reduction of fever, and to counteract collapse. It should be carried out by a doctor or a skilled nurse, and I advise you to accept no nurse as skilled in the technique of the process until you have seen her carry it out on at least one occasion. An ignorant or careless nurse may do much damage, and even perforate the gut.

A child can be placed on the left side, with the hips raised on a pillow; on the back with the buttocks raised; or in the genu-pectoral position. The best position for a baby is on an attendant's lap or on the back with the buttocks raised. In any case a rubber sheet should be so arranged that escaping fluid flows into a basin or other receptacle. The apparatus consists of a soft rubber catheter, size No. 9-12, attached to a rubber tube three to four feet long, with a glass funnel inserted into it. A gum elastic catheter may be used if exceptional care is taken to avoid damage to the mucosa and perforation. A stiff catheter is not necessary if the introduction of fluid is properly managed, although the sigmoid flexure is long and more or less bent on itself, acting as an obstruction to the passage of a tube. The catheter must be well oiled and passed upwards slowly and gently as the fluid flows into the bowel. The pressure of the fluid distends the gut, opens out the folds, and enables the tube to be pushed steadily higher. And it should be so pushed onwards as far as possible within reason, for it is infinitely easier to obtain good results when the fluid is injected into the colon rather than into the rectum, which is liable to evacuate it in a sudden gush as soon as it is distended. The fluid must be allowed to flow in quite slowly under moderate pressure, the funnel being held at a height of one to two feet above the level of the anus. Gentle manipulation of the abdomen during the process assists the flow up the colon. Rapid distension of the rectum is the great cause of failure, for, as I have just stated, it causes the desire to defæcate, and the fluid is simply shot out again. Normally some of the fluid is returned by the side of the catheter from time to time. After the proper amount has been allowed to flow in, the buttocks are compressed together with the hands to keep in the fluid for a short period. Then the tube is detached and the fluid is allowed to return through the catheter. For ordinary cleansing purposes the water should be lukewarm, about 75° F. to 85° F. The amount which may be allowed to flow into an infant's colon at six months of age is one pint, and double the quantity at two or more years of age. The process may be repeated several times if the child is not distressed.

### Rectal Feeding.

The administration of food in the form of nutrient suppositories is valueless and need not be considered. As a matter of fact, rectal feeding is altogether an overrated means of nutrition, and there are comparatively few cases in infants and children in which it is either necessary or advisable. It is more often advisable than necessary at all ages, for the public have an overweening confidence in its value when food cannot be otherwise taken. A

large amount of the benefit is due to the fluid rather than to the nutritive constituents of the injection. Indeed, some physicians maintain that they get as good results from simple saline injections as from nutrient enemata in the treatment of gastric ulcer. In certain affections it is apparently of great value. Thus it is most useful during the first few days after operation for the relief of hypertrophic stenosis of the pylorus and other obstructive diseases of the alimentary tract, and is of assistance in recurrent vomiting and affections in which it is advisable to give the stomach a complete rest. Such rest cannot be entirely obtained by this means, for it must be remembered that the introduction of nutrient material into the lower bowel induces gastric secretion, pain, and vomiting. These symptoms can be relieved sometimes by giving a little milk and bicarbonate of soda by the mouth.

During a course of rectal feeding the rectum and colon should be washed out once daily with saline solution, one drachm of common salt to the pint of water. The rectal feeds should not be given too frequently; they can rarely be continued satisfactorily in babies for more than a week at a time. The best plan is to give from one to two ounces every four hours for two or three days, every six hours for a like period, and then every twelve hours. After an interval of a day or two the course may be repeated, if necessary. An apparatus should be used similar to that recommended for irrigation, but a shorter tube, say one to two feet, is generally quite long enough. The glass funnel is far superior to a syringe of any description, and the importance of giving the feed slowly, and as high up the bowel as possible, should be insisted on. The fluid should be of a temperature of 100° F. to 102° F. If the injection is not retained, it should be at once repeated. The addition of a small quantity of tincture of opium or of chloretone is sometimes recommended under such circumstances; but I am very loath to advise it, as I am convinced that failure depends chiefly on erroneous technique, in not passing the tube sufficiently high for the fluid to flow into the colon.

The continuous method of administration is useful for adults, but rarely available for children, unless comatose, for they are too restless to keep still. A container, surrounded by a hot-water jacket, is fixed at a height of one to two feet (two to four feet for adults) above the level of the anus. From this runs a rubber tube attached to a gum elastic catheter inserted well up the rectum. The tap of the container is turned on so that the nutrient fluid flows into the bowel drop by drop. This is by far the best method, though unfortunately it is so limited in its application.

It has been already stated that rectal feeds are most valuable when they are injected into the colon, for here cane sugar can be converted into grape sugar and a certain amount of starch into dextrin. A considerable amount of digestion and absorption takes place in this part of the gut, especially in the ascending colon. Probably the rectum does not digest food, but can absorb nutritive fluids in solution, possessing a variable capacity for absorption in different children.

Certain fluids and foods are much more readily absorbed than others. Plain water and normal saline solution (0.9 per cent. strength) are readily absorbed in amounts of one to three ounces every one to three hours. Sodium chloride appears to assist the absorption of all kinds of foods. Boyd and Robertson found that pure solution of dextrose in 5.8 per cent. strength is almost entirely absorbed. Commercial dextrose is liable to contain traces of sulphuric acid and prove irritating to the bowel. Cane sugar and dextrin come next in value, and are followed by peptones, but the last are liable to set up diarrhœa. Somatose is valuable, and should be given with salt. Pulverised casein, in the form of plasmon or protene, etc., is not so absorbable. To these substances may be added sanatogen, a pulverised dried casein, containing 5 per cent. of glycerophosphate of soda. But all proteid foods seem to be somewhat poorly absorbed. Even egg albumin, which is commonly used and often recommended, is probably little superior to, though cheaper than, proprietary proteid and peptone preparations. It may be predigested with hydrochloric acid and pepsin. Fat in the yolk of egg can be absorbed to the extent of about 50 per cent., and to a smaller extent when given as emulsified olive oil or milk fat. Unpeptonised milk, starch, and wines are better avoided. Alcohol, when needed, can be added in the form of brandy.

Suitable feeds can now be put before you. First and foremost in value is saline solution, for it supplies the fluid, which is constantly so badly needed, and is most easily absorbed. Or some of these mixtures may be made use of: (a) The white of one egg, salt grs. 10, weak starch and water 3 oz.; (b) Horse serum, *plus* salt grs. 5 to 1 oz.; (c) Yolk of one egg, dextrose ¼ oz., salt grs. 10, water or peptonised milk to 5 oz.; (d) Somatose or sanatogen or plasmon ¼ oz., dextrose or dextrin ¼ oz., salt grs. 10, water or peptonised milk to 3 oz. This may be further enriched by the addition of yolk of one egg and 10 to 30 drops of brandy. If too thick, or it is desirable to give a larger amount of fluid, the mixture can be made up to the required quantity by adding more water.

Leube recommended a nutrient feed composed of scraped raw meat 5 oz., fresh pancreas 2 oz., warm water 5 oz., given in quantities of two to four ounces at a time, and claimed that he got good results. Apart from the difficulty involved in getting fresh pancreas, it is of variable strength, and the mixture is liable to offensive decomposition in the bowel. It is far better to rely on simple saline fluid; solutions of dextrose, dextrin, and cane sugar; the proteid preparations of dried casein, peptones and peptonised milk, and egg albumin; and the fat in yolk of egg, emulsified olive oil, and milk. Even under the most favourable conditions it is impossible to give more than one-fourth of the total quantity of food required by this method, so at best it is only a means of delaying starvation.

*The Reduction of Fever* is brought about by irrigation of the colon with one gallon of water at 95° F.; by enemata of water at 70° F. to 80° F.; by the injection of small quantities of cold or iced water; or by the use of ice suppositories. It is useful in high fever in young children and infants, if there is not much prostration.

*Collapse* should be treated by the injection into the colon of hot water or normal saline at 105° F. to 110° F. The addition of tincture of musk, ½ to 1 drachm, or of brandy, makes the injection still more efficacious. Sometimes it is advisable to add from one to three minims of tincture of opium, *e.g.* in collapse due to severe pain.

*Pain and Tenesmus* can be relieved by iced water, by hot water, by starch solution containing a few minims of tincture of opium, or by suppositories of cocaine grs. ¼. Hot irrigations are useful in colic, but are rarely needed, for a glycerine enema and the external application of heat are generally sufficient.

*Flatulent Abdominal Distension* may be so great as to endanger life by pressing the diaphragm upwards, compressing the lungs and heart, and interfering with respiration and circulation. The passage of a simple rectal tube may afford relief by the evacuation of wind. Enemata are more efficacious, and may be made up of aqua anethi, alone or with an equal quantity of aqua chloroformi; assafœtida ½ drachm to 4 oz. of water; tincture of assafœtida ½ to 2 drachms, in water or starch mucilage.

*Constipation* in babies is treated by a simple cone-shaped piece of paper or ordinary soap, well oiled, introduced into the rectum; soap suppositories, composed of one grain of soap and five minims of oil of theobroma; gluten suppositories or glycerine suppositories. Suppositories are rather less valuable for older children. Or it may be treated at any age by an enema of cold water or normal saline, one half to one ounce under six months of age, one to two ounces for older babies, and four to ten ounces for children; a soap and water enema for older children, but not for babies, as it may be too irritating; an oil enema in about half the above amounts; or a glycerine enema of one-half to one drachm with two to eight drachms of water. Medicated suppositories, though rarely necessary, are sometimes used in the constipation of older children, *e.g.* aloin, ext. belladon., ext. nuc. vom. aa. gr. $\frac{1}{24}$ to $\frac{1}{12}$ night and morning. Their action is probably rather mechanical than due to the drugs incorporated.

For hard fæcal masses in infants give an enema of one to four drachms of warm olive oil, followed in six hours by one of saline solution, one to six ounces. After two years of age a useful injection for impaction consists of ol. terebinth. dr. 1, ol. olivæ drs. 2, yolk of one egg, and warm water to 4 oz. Complete impaction in adults should be treated by a dose of calomel, followed by castor oil, and large injections of thin warm gruel with half an ounce of ol. terebinth, and an ounce of castor oil; by warm olive oil injections; by equal parts of ox gall and water; or by injections of an ounce of Epsom salts, an ounce of olive oil, and 15 oz. of starch mucilage. The quantities must be modified to suit children in the rare cases in which fæcal impaction occurs.

*Colitis and Ileo-Colitis.*—Intestinal lavage at a high temperature, 105° F. to 110° F., once or twice in the 24 hours, is often useful. It should be carried out according to the methods of irrigating the colon, using plain boiled water, saline solution, or a very thin linseed decoction. From 1½ to 8 pints should be used, according to the age of the child. One-third should be injected while introducing the tube, one-third while it is stationary, and one-third during withdrawal. The whole process should take from 20 to 30 minutes. It is most efficacious in the early stages, reduces the colicky pains, relieves the straining, and improves the stools. It checks hæmorrhage, lessens thirst, raises vascular tension and strengthens the infants. The high temperature of the injection facilitates the introduction of the tube by relaxing the contraction of the gut. If there is much hæmorrhage it is advisable to give a further astringent irrigation or an astringent injection to be retained.

The various substances used in irrigation may be grouped as follows:—

1. Simple cleansing solutions.—Water, saline solution, sodium bicarbonate, 1 drachm to the pint.

2. Antiseptics.—Boric acid, sodium salicylate, sodium benzoate, 1 drachm to the pint; naphthol, 3 to 5 grs. to the pint; thymol, 5 to 10 grs. to the pint.

3. Astringents.—Tannin or lead lotion, 1-per-cent. solution; tannic acid ½ drachm, or ext. hamamelis, 1 drachm to the pint, or to 3 or 4 oz. if to be retained; protargol, 2 to 5 grs. to the ounce; argyrol, 1 to 3 grs. per ounce; silver nitrate, ¼ to 1 gr. to the ounce.

5. Sedatives.—Starch, gum arabic, cereal and linseed decoctions.

Silver nitrate is one of the best astringents for use in chronic ulcerative colitis, more valuable in my experience than protargol and other organic silver compounds. Three hours beforehand it should be preceded by an enema of salt and sodium bicarbonate; of the silver solution ½ to 2 pints, or even less, may be injected. If it causes much pain a small dose of tincture of opium or morphia may be given. Such injections should only be given every three or four days, and should be discontinued after three or four unless definitely good results have been obtained. They may cause pain, perhaps severe, and vomiting, but never do harm, though the symptoms may be alarming. Constipation often follows, and, if so, a simple enema must be given in three days' time.

*Prolapse of the rectum* is generally best treated by attention to the regular and normal action of the bowels, and by measures directed to improving the general nutrition of the child. It may be assisted by the introduction of a cone-shaped piece of ice, wrapped in gauze, after the bowels have acted, by small injections of iced water, or by small astringent injections to stimulate the contraction of the gut.

*Threadworms* are commonly attacked per rectum. Thorough irrigation with a 5-per-cent. solution of common salt is as efficacious as most things and free from danger. It washes away the parasites and the mucus in which they live. A large injection to wash out the colon is much more efficacious than small rectal injections. In bad cases in older children and adults stronger drugs may be needed, and recourse can be had to one of the following injections:—(1) Decoct. allii; (2) weak solution of perchloride of iron; (3) santonin, grs. ij., ol. terebinth., drs. ij., mucil acaciæ, 6 oz.; on alternate nights for three times; (4) boric acid, 1 drachm to the pint, to get rid of mucus, followed by solution of perchloride of mercury (1 in 10,000), half a pint every third night; (5) quassia injections. Macerate a small handful of quassia chips for half an hour, decant, and add hot water. After previously emptying the colon by mercury and colocynth pill at night, and a dose of 2 drachms of sodium phosphate in hot water in the morning, inject slowly at 3 P.M. several pints of the fluid at 100° F. Follow the quassia irrigation by an injection of ½ oz. of turpentine in 6 oz. of starch mucilage, anointing the anus and lower part of the rectum with simple ointment to prevent irritation. Repeat the treatment once a week, giving a pill of aloes and asafœtida every night, and keeping the anus clean with 1 in 10,000 perchloride lotion. Much care must be used in treatment by injections of the perchloride on account of the liability to poisonous effects. The injection of decoction of tobacco is a dangerous remedy sometimes used by the poor. Fortunately, these severer measures are rarely needed for children, and they should never be adopted until the simpler ones have been thoroughly tried and failed.

*Objections to enemata and irrigations* must be mentioned. The continued use of soap and glycerine per rectum is liable to cause irritation and catarrh of the mucous membrane. Sometimes mechanical injuries are produced. The effects are lost after a time, so it is necessary to make changes in the variety of enema adopted. Skin rashes are not infrequent. Generally they are erythematous, like the rash of scarlatina, diffuse or confluent; sometimes urticarial. They occur most commonly in young patients who have been given enemata for constipation; they may follow the injection preliminary to operation or the half-pint of salt solution for the cure of threadworms. Obviously they are not due to the soap, for they may follow the injection of plain water. Probably they are the result of the absorption of dissolved fæcal materials. The rash usually comes out in a few hours. It generally begins on the buttocks, spreads down the thighs to the knees, and upwards to the angles of the scapulæ. It may appear on the arms above and below the elbows, and sometimes on the face and neck. Sometimes there is a little fever. The simple erythematous rash generally disappears in less than 24 hours, but the urticarial one may last two or three days. There is no desquamation.

*Drug Treatment.*—It is possible to administer such drugs as quinine, antipyrin, creosote, potassium iodide, etc., in the form of suppositories, but the method is unsatisfactory on account of the impossibility of gauging the amount and rate of absorption. One drug, chloral hydrate, can be given with the greatest advantage by injection in watery solution into the rectum, and I know no more efficacious means of treating convulsions in infancy.

It has been impossible for me to enter more fully into the above method of treatment, and I have limited its application to the more common ailments of everyday practice, leaving out many others which are indeed well worthy of consideration. Yet in other affections the general principles of this treatment are applicable, and you will have no difficulty in devising those modifications suitable for any such disease which may come under your charge.

